# Promotion and Inhibition of Amyloid-β Peptide Aggregation: Molecular Dynamics Studies

**DOI:** 10.3390/ijms22041859

**Published:** 2021-02-13

**Authors:** Satoru G. Itoh, Hisashi Okumura

**Affiliations:** 1 Institute for Molecular Science, National Institutes of Natural Sciences, Okazaki 444-8787, Aichi, Japan; itoh@ims.ac.jp; 2 Exploratory Research Center on Life and Living Systems (ExCELLS), National Institutes of Natural Sciences, Okazaki 444-8787, Aichi, Japan; 3 Department of Structural Molecular Science, SOKENDAI (The Graduate University for Advanced Studies), Okazaki 444-8787, Aichi, Japan

**Keywords:** molecular dynamics simulation, amyloid-β peptide, polyphenol, interface, aggregation, aggregation inhibitor

## Abstract

Aggregates of amyloid-β (Aβ) peptides are known to be related to Alzheimer’s disease. Their aggregation is enhanced at hydrophilic–hydrophobic interfaces, such as a cell membrane surface and air-water interface, and is inhibited by polyphenols, such as myricetin and rosmarinic acid. We review molecular dynamics (MD) simulation approaches of a full-length Aβ peptide, Aβ40, and Aβ(16–22) fragments in these environments. Since these peptides have both hydrophilic and hydrophobic amino acid residues, they tend to exist at the interfaces. The high concentration of the peptides accelerates the aggregation there. In addition, Aβ40 forms a β-hairpin structure, and this structure accelerates the aggregation. We also describe the inhibition mechanism of the Aβ(16–22) aggregation by polyphenols. The aggregation of Aβ(16–22) fragments is caused mainly by the electrostatic attraction between charged amino acid residues known as Lys16 and Glu22. Since polyphenols form hydrogen bonds between their hydroxy and carboxyl groups and these charged amino acid residues, they inhibit the aggregation.

## 1. Introduction

Proteins usually fold correctly and maintain their function in vivo. However, when their concentration increases, such as due to ageing, they aggregate to form oligomers and amyloid fibrils. These protein aggregates are associated with approximately 40 human neurodegenerative diseases [1,2,3]. For example, amyloid-β (Aβ) peptides, which has 40–43 amino acid residues, is associated with Alzheimer’s disease. Huntington’s disease is caused by polyglutamine. Parkinson’s disease and dialysis-related amyloidosis are caused by α-synuclein and β2 microglobulin, respectively. 

The amyloid fibril of Aβ peptides has a cross-β structure [4,5,6,7,8]. There are several experiments that showed the oligomers of Aβ peptides, which are formed before the amyloid fibril, are more toxic than the amyloid fibrils [9,10,11,12]. Atomic-level understanding of the aggregation process has become even more important now. The conformational change during the aggregation and disaggregation process can be clarified by molecular dynamics (MD) simulation. To reveal the aggregation and disaggregation mechanisms of proteins and peptides, several computational studies [13,14] have been performed on the monomeric state [15,16,17,18,19,20,21,22,23,24,25,26], dimerization [27,28,29,30,31,32,33,34,35,36,37,38], oligomerization [39,40,41,42,43,44,45], amyloid fibril elongation [46,47,48,49,50,51,52,53,54,55,56,57,58], amyloid fibril stability [59,60,61,62,63,64,65], and disruption of amyloid fibrils [66,67,68]. 

We review MD simulation studies in environments that enhance and inhibit the Aβ aggregation. Aggregation of Aβ peptides is known to be accelerated at hydrophilic–hydrophobic interfaces, such as membrane surfaces [8,69] and air–water interfaces [70,71]. Oligomerization of peptides at these hydrophilic–hydrophobic interfaces has attracted attention both experimentally [8] and theoretically [24,25]. The inhibition of the Aβ aggregation has also been studied by experimental [72,73] and computational [26,73] approaches. Ono et al. [72,73] found that Aβ oligomerization is inhibited by the polyphenolic compounds. Polyphenols have attracted attention as inhibitors of Aβ aggregation. In the following sections, we first present MD simulations of Aβ peptides at hydrophilic–hydrophobic interfaces [25,45], and then describe a simulation study about the interaction between an Aβ fragment and polyphenols [26].

## 2. Amyloid-β(16–22) Aggregation at Hydrophilic–Hydrophobic Interfaces

In this section, we present MD simulations for aggregation of Aβ(16–22) peptides at hydrophilic–hydrophobic interfaces. This peptide consists of the 16th to 22nd amino acid residues of the Aβ peptide. The amino acid sequence is KLVFFAE. This is a part of the Aβ peptide that contains the hydrophobic amino acid residues and is most responsible for the intermolecular β-sheet of the Aβ amyloid fibril. This part is known to play an important role in the aggregation of Aβ peptides and to form oligomers and amyloid fibrils by itself [74]. This is one of the most frequently studied peptides in simulations because it is shorter and tends to aggregate more than the full-length Aβ peptide [75,76,77,78,79,80].

**Figure 1:**
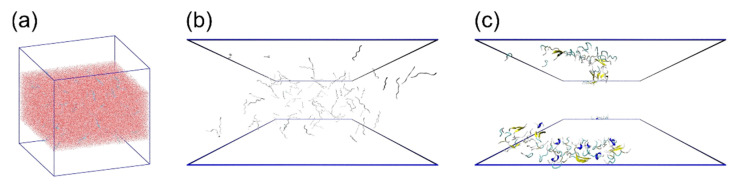
(**a**) Initial conformation of 100 Aβ(16–22) peptides (blue) and water molecules (red) with air–water interfaces. Side views of the (**b**) initial and (**c**) final conformation of Aβ(16–22) peptides. The water molecules are not shown here. The blue frames indicate the air–water interfaces. Reprinted with permission from Reference [45]. Copyright 2020 American Institute of Physics.

We performed MD simulations of Aβ(16–22) peptides at hydrophilic–hydrophobic interfaces [45]. The hydrophilic–hydrophobic interface was modeled here as the interface between the aqueous phase and vacuum phase. First, 162,500 water molecules and 100 Aβ(16–22) peptides were placed in a cubic simulation box with the side length of *L* = 217.69 Å in the range of (1/4) *L* < *z* < (3/4) *L*, as shown in Figure 1a. The Aβ(16–22) peptides were uniformly and randomly distributed, as shown in Figure 1b. All-atom MD simulations were then performed at a temperature of *T* = 310 K to observe the aggregation process of Aβ(16–22) peptides. The simulations were performed using the Generalized-Ensemble Molecular Biophysics (GEMB) program developed by one of the authors (H.O.). We have used this program to simulate several proteins and peptides so far [29,81,82,83,84,85,86,87]. We applied the AMBER parm14SB force field [88] for the peptides. We also used the TIP3P rigid-body water model [89] by adopting the symplectic quaternion scheme [90,91].

As shown in Figure 1a, even though the top and bottom quarters of the simulation box were initially vacuum, a few water molecules evaporated at *T* = 310 K, but most water molecules and Aβ(16–22) peptides did not. Therefore, the interface was maintained spontaneously without any additional force. Other details of the simulation conditions can be found in Reference [45].

**Figure 2:**
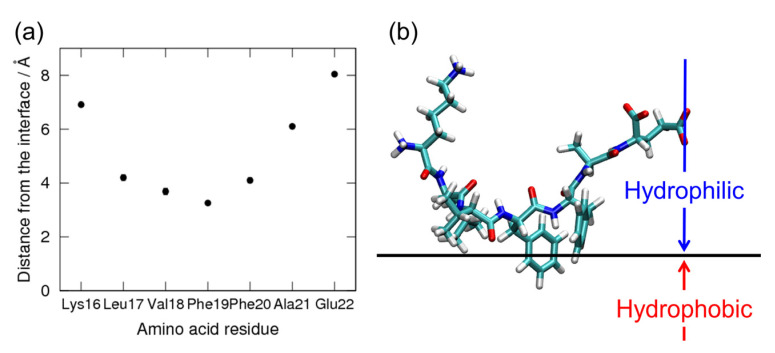
(**a**) Average distance of C_α_ atoms from the interface. (**b**) A typical snapshot of an Aβ(16–22) monomer at the interface. Reprinted with permission from Reference [45]. Copyright 2020 American Institute of Physics.

We observed that the Aβ(16–22) peptides gradually moved to the interface during the simulations. In the end, all Aβ(16–22) peptides moved to the interface, as shown in Figure 1c. This is because Aβ(16–22) peptides have both hydrophilic (Lys, Glu) and hydrophobic (Leu, Val, Phe, Ala) amino acid residues, and the hydrophilic residues tend to exist in water, while the hydrophobic residues tend to exist in the hydrophobic region, as shown in Figure 2. Figure 2a shows an average distance between the C_α_ atom of each residue and the interface, and Figure 2b shows a typical snapshot of an Aβ(16–22) monomer at the interface. The reason why full-length Aβ peptides are abundant on the surface of cell membranes in vivo is because this is the hydrophilic–hydrophobic interface, and Aβ peptides consist of both hydrophilic and hydrophobic amino acid residues. In other words, Aβ peptides are amphiphilic molecules, like surfactants. Therefore, the concentration of Aβ peptides increase at the interface, and they tend to aggregate there.

**Figure 3:**
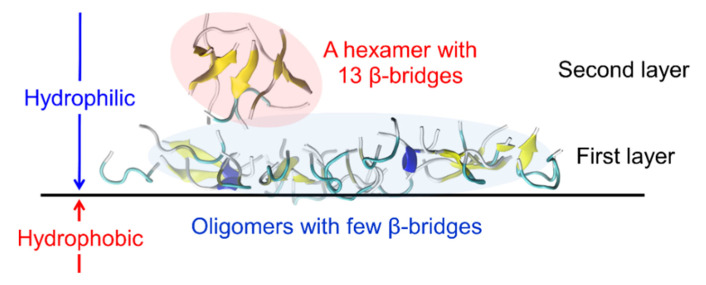
Snapshot of oligomers that had a few β-bridges in the first layer and a hexamer with 13 β-bridges in the second layer. Reprinted with permission from Reference [45]. Copyright 2020 American Institute of Physics.

**Figure 4:**
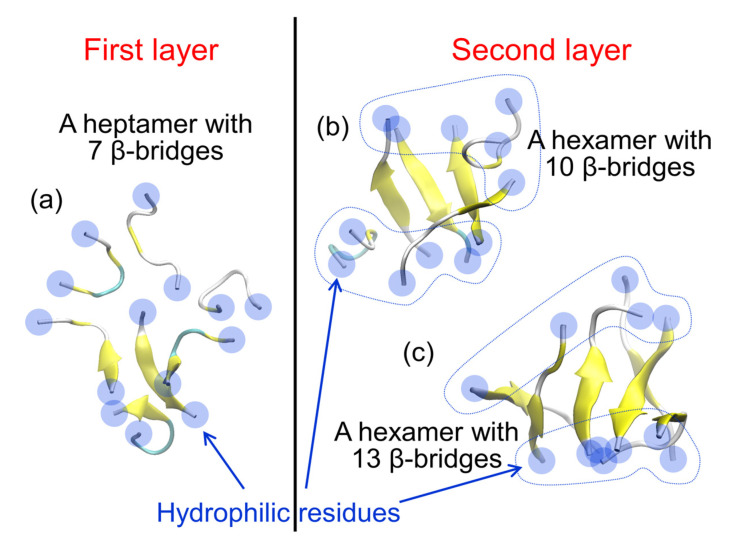
Typical snapshots of Aβ(16–22) oligomers (**a**) found in the first layer and [(**b**) and (**c**)] found in the second layer. Reprinted with permission from Reference [45]. Copyright 2020 American Institute of Physics.

Typical aggregates of the Aβ(16–22) peptides at the interface are shown in Figure 3. We found that the aggregates consist of two layers. The first layer is close to the interface, and the second layer is on the aqueous side. In the first layer, most of the Aβ(16–22) peptides are either monomers that do not form hydrogen bonds with each other or aggregates with only one or two β-bridges. In the second layer, most of the seven amino acid residues of the Aβ(16–22) peptide form intermolecular β-sheets. This peptide has a negatively charged Lys at the N-terminus and a positively charged Glu at the C-terminus. The electrostatic attraction between these residues tends to form antiparallel intermolecular β-sheets. In the second layer, the antiparallel intermolecular β-sheet is well formed, and the hydrophilic amino acid residues (Lys and Glu) at both ends are aligned along the edge of the oligomer, covering the hydrophobic amino acid residues (Figure 4b,c). This makes the oligomer more soluble in water. This is the reason why the oligomer with more intermolecular β-bridges exist in the second layer. In the first layer, on the other hand, the hydrophilic amino acid residues are not well aligned, and the hydrophobic residues are not covered by the hydrophilic residues (Figure 4a). The hydrophilic residues are sometimes located in the center of the oligomer, and the hydrophobic residues are exposed along the edge of the oligomer. These oligomers are, therefore, present in the first layer, exposing the hydrophobic residues to the hydrophobic region, as shown in Figure 2b.

Jean et al. experimentally found that amyloid-forming peptides adsorb in layers for up to about 80 nm from the interface [71]. Our simulations that show the layer formation agree with these experimental results. In addition, our simulations suggest that the formation of intermolecular β-sheets may be promoted mainly in the second (or higher) layer.

## 3. Conformation of an Amyloid-β 40 Peptide at a Hydrophilic–Hydrophobic Interface

We now describe the conformations of a full-length Aβ peptide, Aβ40, that consists of 40 amino acid residues at a hydrophilic–hydrophobic interface. The amino-acid sequence of Aβ40 is DAEFRHDSGYEVHHQKLVFFAEDVGSNKGAIIGLMVGGVV. It is known that two intermolecular β-sheet structures are formed in the amyloid fibril of the Aβ peptides [92]. These β-sheets are formed at residues 10−22 (β1) and residues 30−40 (β2). Most of the β1 and β2 regions are composed of hydrophobic residues. 

We performed MD simulations for an Aβ40 molecule in a system with hydrophilic–hydrophobic interfaces [25]. The hydrophilic–hydrophobic interface was prepared again by removing water molecules located in the lower half of a cubic simulation box. The N-terminus and C-terminus of the Aβ40 molecule were not capped here. For comparison, MD simulations of an Aβ40 molecule in bulk water were also performed. Other simulation details can be found in Reference [25].

**Figure 5:**
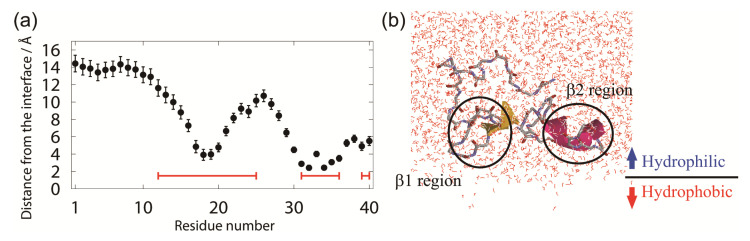
(**a**) Average distance of each C_α_ atom of the Aβ peptide from the interface. Red lines show the residues that bound to the lyso-GM1 micelle in the experiments [93]. (**b**) A typical snapshot of the Aβ40 peptide at the interface. Reprinted with permission from Reference [25]. Copyright 2019 American Chemical Society.

We observed that Aβ40 existed at the hydrophilic–hydrophobic interface as well as Aβ(16–22) peptides. To see the conformations of Aβ40 at the interface, we calculated the average distance of C_α_ atoms from the interface, as shown in Figure 5a. When this value is positive (negative), the C_α_ atom is in the hydrophilic (hydrophobic) region. We can see from this figure that Aβ40 has an up-and-down shape. This result agrees well with previous NMR experiments on Aβ40 conformation on lyso-GM1 micelles [93]. It is known that Val12−Gly25, Ile31−Val36, and Val39−Val40 of Aβ40 bind to lyso-GM1 micelles. The β1 region almost consists of the residues Val12−Gly25. The β2 region includes Ile31−Val36 and Val39−Val40. Therefore, the β1 and β2 regions bind to the lyso-GM1 micelles. It was also reported that the Aβ monomer has an up-and-down shape at the hydrophilic–hydrophobic interface. Figure 5b shows a typical conformation at the interface. In this conformation, the β1 and β2 regions bind to the interface. The N-terminal region and the linker region between β1 and β2 are in the aqueous solvent.

**Figure 6:**
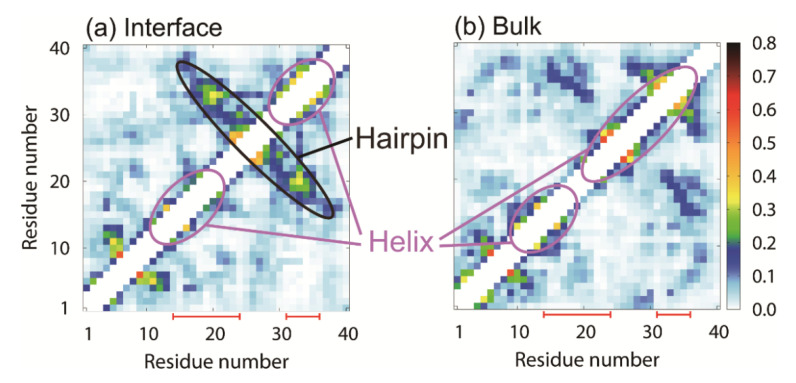
Contact probabilities of C_α_ atoms (**a**) in the presence of the interface and (**b**) in the absence of the interface. Red solid lines correspond to the residues with helix structures in the experiments with the lyso-GM1 [93]. Reprinted with permission from Reference [25]. Copyright 2019 American Chemical Society.

In order to investigate the effects of the interface on Aβ40 structures, we calculated contact probabilities of C_α_ atoms from our MD simulations. Figure 6a,b shows the contact probabilities with the interface and without the interface, respectively. The β1 and β2 regions formed helix structures at the interface. This is consistent with the experimental results on the lyso-GM1 micelle [93]. By forming the contacts between the β1 and β2 regions, not only the helix structures but also a hairpin structure was formed. In the bulk water, both regions had helix structures as well as at the interface, as shown in Figure 6b. However, the probability of the hairpin structure in the bulk water was lower than that at the interface. The difference in the forming ability of the hairpin structure between the interface and in the bulk water would cause a difference in the ability to form the oligomers. In fact, we reported that a β-hairpin structure facilitates the formation of intermolecular β-sheet structures with other Aβ fragments [30,42]. That is, the β-hairpin structure accelerates the oligomer formation with the intermolecular β-sheet structure. Several experimental and computational studies, as well as ours, showed that the β-hairpin structure played an important role in oligomer formation [94,95]. As we described in the previous section, because they have both hydrophilic and hydrophobic residues, the Aβ40 peptides gather at the hydrophilic–hydrophobic interface. The increase in the Aβ40 concentration promotes the aggregation at the interface. Not only the high concentration but also the structure of Aβ40 itself accelerates the aggregation.

**Figure 7:**
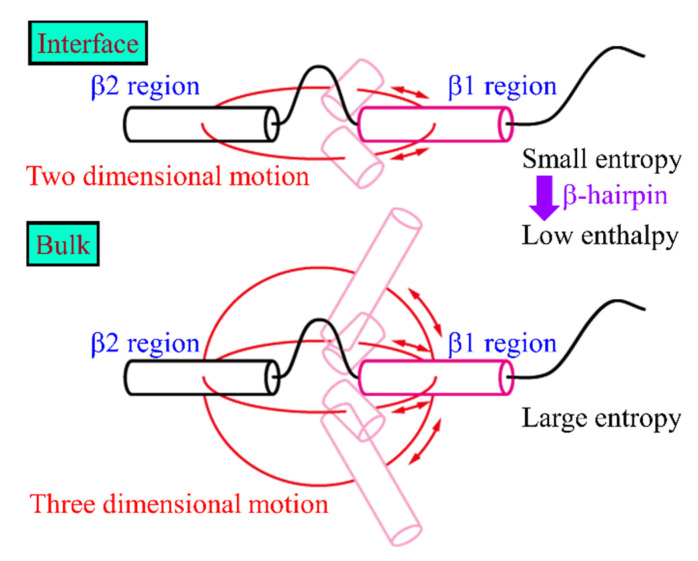
Schematic illustration of the Aβ40 peptide at the interface and in the bulk water. Reprinted with permission from Reference [25]. Copyright 2019 American Chemical Society.

We can consider why the β-hairpin structure is formed at the interface more easily than in the bulk water as follows. The β1 and β2 regions get trapped at the interface, as shown in Figure 5. These regions can move only at the interface, as shown in Figure 7. Thus, the relative motion of the β1 region to the β2 region is suppressed in two dimensions. In the bulk water, on the other hand, the β1 region can move in three dimensions. By taking various conformations, the entropy increases in the bulk water. At the interface, however, the entropy increase is suppressed because of the two-dimensional motion. Therefore, lower enthalpy conformations are preferred to decrease the free energy. The hydrogen-bond formation between the β1 and β2 regions decreases the enthalpy. The β-hairpin structures are, thus, formed.

**Figure 8:**
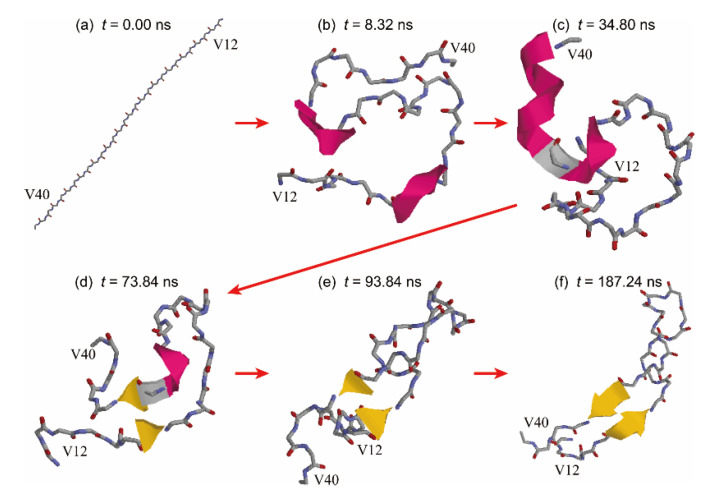
Time-series snapshots of Aβ40 at the interface at (**a**) *t* = 0.00 ns, (**b**) *t* = 8.32 ns, (**c**) *t* = 34.80 ns, (**d**) *t* = 73.84 ns, (**e**) *t* = 93.84 ns, (**f**) *t* = 187.24 ns. Residues D1−E11 was omitted for the sake of clarity. Reprinted with permission from Reference [25]. Copyright 2019 American Chemical Society.

To explain this mechanism in more detail, time-series snapshots are presented in Figure 8. The initial Aβ40 conformation was a fully extended structure (Figure 8 a). The β1 and β2 regions first formed helix structures, as shown in Figure 8b. These regions stably bound to the interface and moved only at the interface. The helix structure in the β1 region was then broken, as shown in Figure 8c. The extended β1 region approached the β2 region, and a β-bridge was formed between these regions (Figure 8d). The β-bridge kept being formed, but the helix structure in the β2 region was broken, as shown in Figure 8e. The β-hairpin structure was finally formed, as shown in Figure 8f. In this way, hydrogen bonds between the β1 and β2 regions were formed step-by-step, changing the helix structures to the extended structures.

## 4. Interaction between Amyloid-β(16–22) and Polyphenols

**Figure 9:**
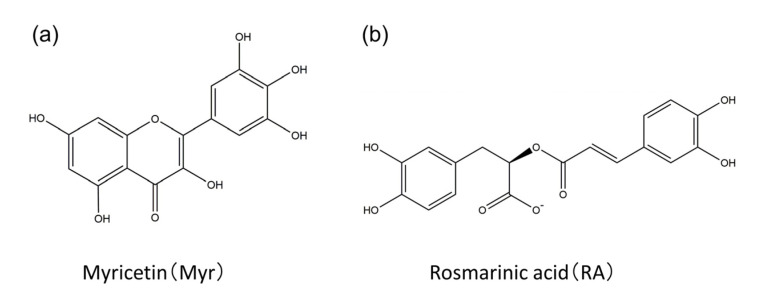
Chemical structures of the (**a**) myricetin (Myr) and (**b**) rosmarinic acid (RA). Reprinted with permission from Reference [26]. Copyright 2020 Elsevier.

Finally, we present MD simulations of Aβ(16–22) peptides and polyphenols [26]. Polyphenols are known to inhibit the aggregation of Aβ peptides and have attracted attention as candidate molecules for drugs against Alzheimer’s disease. There are several types of polyphenols. However, according to recent experiments, myricetin (Myr) and rosmarinic acid (RA) are the most effective polyphenols in inhibiting Aβ aggregation (Figure 9) [73]. However, the mechanism by which these compounds inhibit the aggregation of Aβ is not well understood.

**Figure 10:**
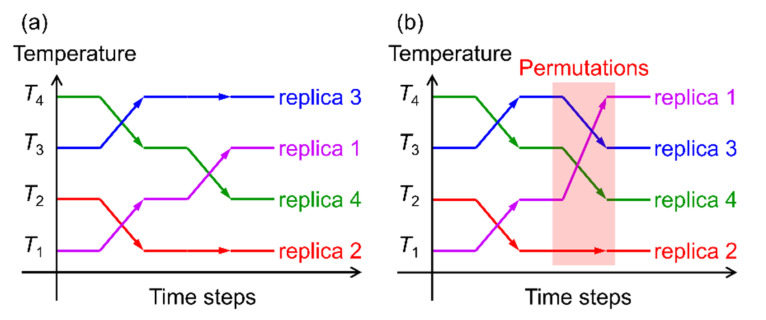
Illustration of the (**a**) replica-exchange method and (**b**) replica-permutation method. In the replica-exchange method, temperatures are exchanged between two replicas, whereas, in the replica-permutation method, temperatures are permuted among three or more replicas.

We performed replica-permutation MD simulations of a system containing an Aβ(16–22) peptide and these polyphenols [26]. The replica-permutation method [96] is one of the generalized-ensemble algorithms [97,98,99] developed by the authors and is an improved alternative to the replica-exchange method [100,101]. In these methods, several copies of the system (called replicas) are prepared, and different temperatures are assigned to the replicas. In the replica-exchange method, the temperatures are exchanged between two replicas during the simulation, as shown in Figure 10a. On the other hand, in the replica-permutation method, the temperatures are permuted among three or more replicas, as shown in Figure 10b. In this case, we use the Suwa-Todo method [102], which is the most efficient Monte Carlo method and is employed in several generalized ensemble algorithms [19,96,103,104,105,106]. The replica-permutation method provides statistically more reliable data on the structure of biomolecules [96,103]. There are several versions of the replica-permutation method [19,103,104,105], such as the Hamiltonian replica-permutation method [19] and isobaric-isothermal replica-permutation method [104], but the original replica-permutation method [96] was used here to change temperatures.

Using this method, we performed all-atom MD simulations of systems containing one Aβ(16–22) peptide and one polyphenol molecule. We employed two polyphenols, Myr and RA, and observed how these polyphenols bound to the Aβ(16–22) peptide. The GEMB program was used again for the simulations. Each system consists of one Aβ(16–22) peptide, one polyphenol molecule, and water molecules. A Na+ ion was added as a counter ion for the RA system. To reduce the effect of the N-terminal and C-terminal electric charges of the Aβ(16–22) peptide, the N-termini and C-termini were blocked by acetyl and N-methyl groups, respectively. Details of the other simulation conditions can be found in Reference [26].

**Figure 11:**
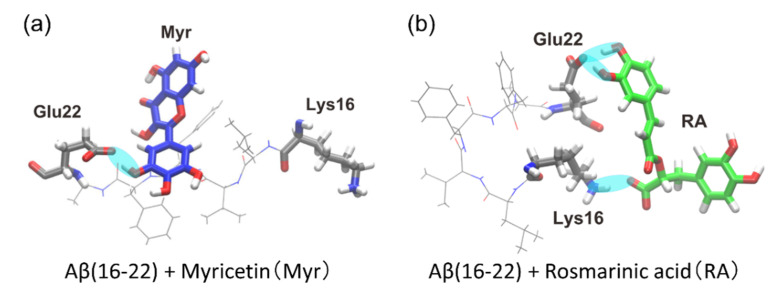
Typical snapshots of the (**a**) Myr system and (**b**) RA system that have hydrogen bonds with the Aβ(16–22) peptide. Cyan ovals indicate hydrogen bonds. Reprinted with permission from Reference [26]. Copyright 2020 Elsevier.

As a result, we observed that polyphenols bound to the Aβ(16–22) peptide, as shown in Figure 11. The cyan ovals between the Aβ(16–22) peptide and each polyphenol indicate the hydrogen bond between them. In the Myr system, the carboxyl group (-COO) of Glu22 often bound to the hydroxy group (-OH) of Myr, as shown in Figure 11a. In the RA system, the carboxyl group of Glu22 frequently bound to the hydroxy group of RA, and the amine group (-NH3) of Lys16 formed a hydrogen bond with the carboxyl group of RA, as shown in Figure 11b.

**Figure 12:**
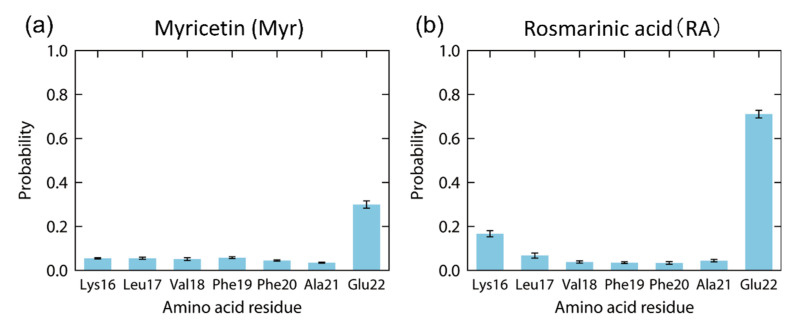
Contact probability of each amino acid residue of the Aβ(16–22) peptide with (**a**) myricetin and (**b**) rosmarinic acid at 300 K. Reprinted with permission from Reference [26]. Copyright 2020 Elsevier.

The probabilities that these polyphenols have contacts with the Aβ(16–22) peptide were calculated, as in Figure 12. In the Myr system, Myr mainly binds to Glu22 with the highest probability of 30%, as in Figure 12a. However, the other residues of the Aβ(16–22) peptide have much fewer interactions with Myr. In the case of the RA system, high contact probabilities were found at two residues, Glu22 with 71% and Lys16 with 17%, as shown in Figure 12b. On the other hand, the hydrophobic residues (Leu, Val, Phe, and Ala) have low contact probabilities of less than 7%. As shown in the previous section, when Aβ(16–22) peptides aggregate, they form antiparallel β-sheets due to the electrostatic attraction between the carboxyl group of Glu22 and the amine group of Lys16. Therefore, we can expect that the aggregation of Aβ(16–22) peptides is inhibited by binding of Myr and RA to the side chains of Glu22 and Lys16, as shown in Figure 11.

**Figure 13:**
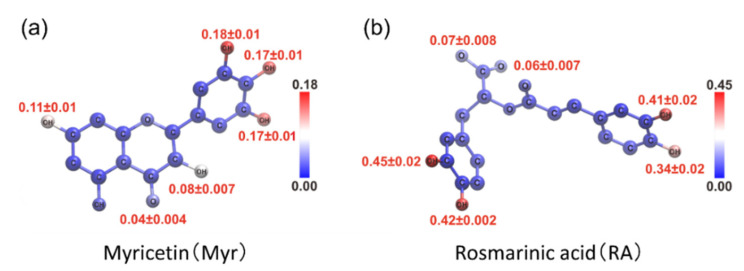
Color mapping of contact probability of the (**a**) myricetin and (**b**) rosmarinic acid atoms with the Aβ(16–22) peptide at 300 K. Reprinted with permission from Reference [26]. Copyright 2020 Elsevier.

In order to clarify which atoms of polyphenols contribute to the interaction with the Aβ(16–22) peptide, we also calculated the contact probability of each atom of polyphenols, as shown in Figure 13. In both Myr and RA systems, multiple adjacent hydroxy groups around six-membered rings have high contact probabilities with the Aβ(16–22) peptide. In RA, the carboxyl group also makes some contacts with the Aβ(16–22) peptide. These atoms in polyphenols play an important role in inhibiting the aggregation of the Aβ(16–22) peptides.

## 5. Conclusions

We presented molecular dynamics (MD) simulations of an Aβ40 peptide and Aβ(16–22) fragments to study the aggregation process and the binding structures of drug candidates that inhibit aggregation. Aβ40 peptides and Aβ(16–22) fragments have both hydrophilic and hydrophobic amino acid residues, and, thus, tend to exist at hydrophilic–hydrophobic interfaces, such as cell membrane surfaces and air-water interfaces. The high concentration of the peptides is, therefore, one reason for the acceleration of aggregation at the interface. In addition, Aβ40 formed a hairpin structure by getting the β1 and β2 regions closer to each other. Such a hairpin structure was rarely formed in the bulk water. This is because the β1 and β2 regions can move only at the interface, and the entropy at the interface becomes smaller than in the bulk water. In order to decrease the free energy, it is required to decrease the enthalpy. Lower enthalpy is realized by hydrogen-bond formation between the β1 and β2 regions. Thus, the β-hairpin structure is formed. It is known that the β-hairpin structure plays an important role in oligomer formation [30,42,94,95]. The β-hairpin structure accelerates the formation of an oligomer with the intermolecular β-sheet structure. Since the hairpin structure is readily formed at the interface, the oligomer is formed more easily at the interface than in the bulk water.

We also review how polyphenols such as myricetin and rosmarinic acid interact with Aβ(16–22) peptides and inhibit the aggregation. The aggregation of Aβ(16–22) peptides is caused mainly by the electrostatic attraction between charged amino acid residues (Lys16 and Glu22). The polyphenols are expected to inhibit the aggregation by forming hydrogen bonds between their hydroxy and carboxyl groups and these charged amino acid residues. No MD simulation has been performed for a negative control, such as a mutated Aβ(16–22) peptide at Lys16 or Glu22. Such a simulation may be another interesting target for a future project.

In this way, MD simulations can identify which atoms are important for aggregation and aggregation inhibition. Recent advances in supercomputers have made it possible to simulate large systems and long-lasting phenomena that were previously impossible to simulate on a computer. In the future, MD simulations could be used to design useful molecules for the treatment of neurodegenerative diseases, such as Alzheimer’s disease. It is hoped that MD simulations will become a tool for developing treatments for these diseases. We also think that such simulation-based predictions should be made in conjunction with experiments to ensure the results.
